# Community acquisition of β-lactamase producing *Enterobacteriaceae* in neonatal gut

**DOI:** 10.1186/1471-2180-13-136

**Published:** 2013-06-17

**Authors:** Charu Kothari, Rajni Gaind, Laishram Chandreshwor Singh, Anju Sinha, Vidya Kumari, Sugandha Arya, Harish Chellani, Sunita Saxena, Monorama Deb

**Affiliations:** 1 Department of Microbiology, VMMC and Safdarjung Hospital, New Delhi 110029, India; 2 National Institute of Pathology, Indian Council of Medical Research, New Delhi 110029, India; 3 Indian Council of Medical Research, New Delhi 110029, India; 4 Department of Paediatrics, VMMC and Safdarjung Hospital, New Delhi 110029, India

**Keywords:** ESBL, AmpC, Carbapenemases, Neonates, Antibiotic resistance, *Enterobacteriaceae*, Fecal carriage, Gut colonization

## Abstract

**Background:**

Commensal flora constitutes a reservoir of antibiotic resistance. The increasing variety of β-lactamases and the emergence of Carbapenem resistant *Enterobacteriaceae* (CRE) in community, raise concerns regarding efficacy of β-lactams. It is important to know the exact load of antibiotic resistance in the absence of any antibiotic selection pressure including via food and water.

In the present study gut colonization in neonates with no direct antibiotic pressure was used as a model to evaluate β-lactam resistance in the community.

**Results:**

In this prospective study, 75 healthy, vaginally delivered, antibiotic naive, breast fed neonates were studied for gut colonization by Extended spectrum β-lactamases (ESBL), AmpC β-lactamases hyperproducing *Enterobacteriaceae* and CRE on day 0, 21 and 60. Total 267 *Enterobacteriaceae* were isolated and *E.coli* was the predominant flora. ESBL, AmpC and coproduction was seen in 20.6%, 19.9% and 11.2% isolates respectively. ESBL carriage increased threefold from day 1 to 60 showing predominance of CTX-M group 15 (82.5%), *ampC* genes were heterogeneous. Colonization with CRE was rare, only one baby harboured *Enterobacter sp* positive for *kpc-2*. The reservoirs for these genes are likely to be mother and the environment.

**Conclusions:**

Data strongly suggests that in absence of any antibiotic pressure there is tremendous load of antibiotic resistance to β-lactam drugs. Wide spread presence of ESBL and AmpC can drive rapid emergence and dissemination of CRE. This is the first report from India which depicts the smaller picture of true antibiotic pressure present in the Indian community.

## Background

The rapid dissemination of antibiotic resistance in bacteria constitutes a major public health concern worldwide. Selective pressure mediated by the intensive use of antibiotics (both human and non-human) and several mechanisms for genetic transfer could have contributed to the rapid dispersal of antibiotic resistance in the community [1]*.*

Antibiotics target both pathogenic bacteria as well as normal commensal flora, represented by skin, gut, and upper respiratory tract [2]*.* Current strategies to monitor the presence of antibiotic resistance in bacteria mainly rely upon examining resistance in pathogenic organisms and involve only periodic cross-sectional evaluations of resistance in the commensal flora [3,4]*.* Resistance amongst the commensal flora is a serious threat because a very highly populated ecosystem like the gut may at later stage be source of extra intestinal infection which may spread to other host or transfer genetic resistance element/s to other members of micro-biota including pathogens [5]*.* Despite this, there is paucity of data regarding the dynamics of antibiotic resistance in commensals.

β-lactam antibiotics are the most commonly used antibiotics in community as well as hospitals*.* They are generally characterized by their favorable safety and tolerability profile as well as their broad spectrum activity [6]*.* The ever increasing variety of β-lactamases raises serious concern about our dependence on β-lactam drugs. Rapidly emerging β-lactamases include diverse ESBL, AmpC β-lactamases, and carbapenem-hydrolyzing β-lactamases*.* ESBL producing *Enterobacteriaceae* were initially associated with nosocomial infections, however, recent studies indicate significant increase in the community isolates [7]*.* The risk posed by community circulation of the multidrug resistant bacteria is emphasized by the high concentration of ESBL in the community as well as the hospital onset intra-abdominal infections [8].

The rapid dissemination of ESBL’s in community may drive excessive use of carbapenems. The recent report of Carbapenem resistance due to dissemination of NDM-1 β-lactamase producing bacteria in the environmental samples and key enteric pathogens in New Delhi, India may have serious health implications [9]*.* Several studies have been conducted to assess the risk factors associated with colonization and infection caused by ESBL producing *Enterobacteriaceae*, which include antibiotic use, travel, contact with healthcare system and chronic illness [10,11]*.*

Gut colonization in neonates with no direct antibiotic pressure were used as a model to evaluate β-lactam resistance in the community in absence of selection pressure.

## Methods

### Design overview, setting, and participants

In this prospective study all low birth weight neonates (LBW) (≥1500 to <2500 g) born at the Safdarjung Hospital, New Delhi, India (2009–2011) were eligible and enrolled to study ‘Effect of Probiotic VSL#3 on prevention of sepsis during 0–2 month period’. This is double blind study in which neonates were randomized to receive either VSL# 3 for 30 days in intervention group or physically similar preparation (Maltdextrin) in control group. The consent was obtained from parents of each neonate prior to enrolment. The stool samples from 75 randomly selected LBW neonates were used to study gut colonization with ESBL, AmpC and carbapenemase producing *Enterobacteriaceae*. The inclusion criteria were vaginally delivered, healthy and exclusively breast fed LBW neonates. The exclusion criteria were gross congenital malformations, hospitalization, prematurity, predisposing factors for sepsis, antibiotics use by mother during pregnancy and neonates during study period. After discharge from the hospital, trained field workers visited the newborns for probiotic supplementation, collection of stool sample and related complications up to 60 days of life. The study was duly approved by ethical committee of Safdarjung Hospital.

### Study of colonization by *Enterobacteriaceae*

Stool samples were collected on Day (D) 1, 21 and 60, serially diluted and plated on McConkey agar without antibiotic to study dominant gut flora. D1 sample is the first stool passed after birth (meconium). Different colony types of gram negative bacteria which were judged to differ in morphology (size, shape, consistency and colour) from each sample were enumerated separately and identified using conventional biochemical tests.

### Phenotypic assessment and molecular characterization of antimicrobial susceptibility

All *Enterobacteriaceae* isolated were screened for ESBL using disk diffusion and Etest methods (AB BIODISK, Solna, Sweden) and plasmid mediated AmpC or hyperproduction using AmpC disc test [12]. In 27 randomly selected neonates *Enterobacteriaceae* were characterised for *ESBL* (*bla*_*TEM*_, *bla*_*SHV*_ (self designed, Table 1), *bla*_*CTX-M*_ [group1, 2, 8, 9 and 25]) [13] and *ampC* (*MOX*, *CIT*, *DHA*, *ACC*, *EBC*, and *FOX*) [14] genes.

**Table 1:** Primers used for detection of TEM, SHV and Carbapenemase genes

**Primers**	**Primer Sequence (5′ to 3′ direction)**	**Annealing**	**Amplicon size**
		**Temperature (°C)**	**(bp)**
TEM	FP- ATG AGT ATT CAA CAT TTC CG	50	858
	RP- CCA ATG CTT AAT CAG TGA GG		
SHV	FP- ATG CGT TAT ATT CGC CTG TG	58	862
	RP- AGC GTT GCC AGT GCT CGA TC		
KPC-1	FP- AGC CGT TAC AGC CTC TGG AG	55	1351
	RP- GAT GGG ATT GCG TCA GTT CAG		
KPC-2	FP- CAC TGT ATC GCC GTC TAG TTC	55	812
	RP- TGT GCT TGT CAT CCT TGT TAG		
NDM-1	FP- CGACGATTGGCCAGCAAATG	58	551
	RP- ACTTGGCCTTGCTGTCCTTG		
IMP	FP- TTGAAAAGCTTGATGAAGGCG	58	616
	RP- ACCGCCTGCTCTAATGTAAG		
VIM	FP- TTGACCGCGTCTATCATGGC	58	762

### Carbapenemase screening

All neonates were screened for gut colonization by carbapenem resistant *Enterobacteriaceae* (CRE) using 2-step broth enrichment method incorporating 10 μg meropenem disc [15]. Suspected CRE isolates with resistance to any one carbapenem [16] i.e. ertapenem (Minimum inhibitory concentration (MIC) > 0.25 μg/ml), imipenem and meropenem (MIC >1 μg/ml) by Etest (bioMérieux, France) were tested for metallo- β lactamase (MBL) production using IPM/ethylenediamine tetra-acetic acid (EDTA) Etest and for non-metallo-carbapenemase (NMC), especially KPC, production by the modified Hodge test (MHT) [16]. PCR for *VIM*, *IMP*, *KPC* and *NDM-1* genes (self designed, Table 1) was performed for confirmation.

### Sequence analysis

All isolates found to carry *ESBL*/*ampC* or carbapenemase gene were further confirmed by sequencing. Sequencing was performed as per manufacturer’s guidelines in 3130×l genetic analyser (Applied Biosystems, Foster city, California). Further the nucleotide and deduced amino acid sequences were analyzed and compared with sequences available in Gene bank at the National centre of Biotechnology Information (NCBI) web site (http://www.ncbi.nlm.nih.gov/).

## Results

### Gut colonization pattern of *Enterobacteriaceae* and distribution of ESBL and AmpC β -lactamases in healthy low birth weight Neonates (1–60 days)

On D1, 65.3% of babies were colonized with *Enterobacteriaceae* with no significant increase on D60. The predominant flora was *E. coli* on day 1, 21 and 60 followed by *Klebsiella pneumoniae* (Table 2).

**Table 2:** **Distribution of *Enterobacteriaceae *and associated ESBL and **^**AmpC β-**^**lactamases in Neonates**

	**Total**	**Day 1**	**Day 21**	**Day 60**
		**(N = 75)**	**(N = 75)**	**(N = 75)**
		**No. (%)**	**No. (%)**	**No. (%)**
**Babies colonized with a least one species**		49 (65.3)	48 (64)	53 (70.6)
**No of babies colonized with at least one ESBL producing isolate**		7/49 (14.3)	13/48 (27.1)	22/53 (41.5)*
**Total *Enterobacteriaceae *strains #**	**267**	**79**	**88**	**100**
*E.coli*	219	69 (87.3)	67 (76.1)	83 (83)
*Klebsiella pneumoniae*	27	3 (3.8)	13 (14.8)	11 (11)
*Enterobacter sp*	14	2 (2.5)	7 (8)	5 (5)
*Citrobacter sp*	5	4 (5.1)	0	1 (1)
*Salmonella. Typhi*	2	1 (1.3)	1(1.1)	0
Total ESBL	55 (20.6)	7 (8.9)	17 (19.3)	31 (31)**
Total *AmpC* (N = 39)	53 (19.9)	16 (20.3)	12 (13.6)	25 (25)***
Co-Production of ESBL and *AmpC*	30 (11.2)	5 (6.3)	9 (10.2)	16 (16)****

Note: Data represents *Enterobacteriaceae* isolates from gut of 75 healthy Low birth weight (LBW) neonates on Day 1, 21, 60 of birth.

All Figures in parentheses represent percentages.

# Some babies had more than one morphologically and biochemically distinct isolates.

*p value 0.005 **p value 0.001 ***p value 0.2 ****p value 0.05 when compared to Day 1.

Overall ESBL and AmpC production was 20.6% and 19.9% respectively. The total isolates positive for either AmpC and or ESBL were 29.2% (78/267). The predominant phenotypes were co-producers (30/267, 11.23%), followed by only ESBL (25/267, 9.4%) and AmpC (23/267, 8.6%) isolates. Both no. of babies colonized with at least one ESBL producing isolate and ESBL rate amongst *Enterobacteriaceae* increased three fold (p value 0.005 and 0.001 respectively) from day 1 to day 60, irrespective of associated AmpC production (Table 2).

### Characteristics of ESBL and AmpC β - lactamases in *Enterobacteriaceae* isolates from 27 randomly selected neonates

The three stool samples from 27 neonates generated 88 gram negative bacilli which included *E.coli* (N = 74), *Klebsiella pneumoniae* (N = 11), *Citrobacter freundii* (N = 2) and *Enterobacter aerogenes* (N = 1). *CTX-M-15* is predominant *ESBL, TEM-136, TEM-149, SHV-28* and *CTX-M-*8 was seen in single isolates. In contrast, the *ampC* was diverse and included *DHA* (N = 5), *CMY-2* (N = 3), *CMY-1* (N =2), *MOX* (N = 2) and *FOX* (N = 1) (Table 3).

**Table 3:** **Molecular Characterization of ESBL & AmpC β-lactamases in *Enterobacteriaceae *isolated (N = 88) from 27 randomly selected neonates**

**Phenotype**	**No. strains**	**ESBL**				**AMPc**						
		**bla-TEM**	**ESBL TEM**	**SHV**	**CTX-M**	**DHA**	**CMY-1**	**CMY-2**	**LAT**	**MOX**	**BIL**	**FOX**
**E + A+**	7	7	2*	1**	4**#**	2		3		2		1
**E + A-**	10	10			10							
**E-A+**	5	5				3**##**	2					
**E-A-**	66	PCR not performed for strains with cefotaxime, ceftazimime zone diameter ≥ 28 and ≥ 23 respectively and phenotypic test negative for ESBL and AmpC**16**										

Note: Sequencing results *Tem 136, Tem 149; **SHV28. E = ESBL, A = AmpC, - = Negative, + = Positive.

^#^*ESBL* and *AMPc* genes were mainly isolated in *E.coli* except one *Klebsiella pneumoniae* having both CTX-M as well as MOX gene.

^##^ One Citrobacter showed the presence of DHA gene.

### Colonization by carbapenem resistance *Enterobacteriaceae* in the neonates

Total 225 stool samples from 75 enrolled babies were screened for CRE 2-step broth enrichment method incorporating 10 μg meropenem disc. Gram negative colonies were isolated from 22 stool samples, which yielded 29 *Enterobacteriaceae* isolates that were presumed to be CRE. Phenotypic test for MBL was negative, MIC of 28 suspected CRE ranged from 0.012-0.5 μg/ml, 0.016-0.125 μg/ml and 0.094-0.38 μg/ml for ertapenem, meropenem and imipenem respectively. However, one isolate of Enterobacter *aerogenes*. was positive for MHT having the MIC of > 32 μg/ml for ertapenem, meropenem and imipenem. Presence of *kpc-2* gene was confirmed by PCR using gene specific primers.

## Discussion

In the present report we have investigated the β-lactam resistance pattern amongst *Enterobacteriaceae* in gut flora of neonates (1–60 days) by enrolling babies using various selection criteria so as to avoid any possible source of antibiotic selection pressure. Acquisition of resistance through food and water was also ruled out as neonates were exclusively breast fed. Compliance was ensured through household follow up by trained field workers upto D60 of life.

The present study shows that majority of the babies were colonized by D1. With the acquisition of mother’s flora the babies are equally likely to get the antibiotic resistance strains. Our data revealed that overall there was nearly 87% (232/267) resistance to the ampicillin by D60 in *Enterobacteriaceae*. The overall rate of ESBL was 20.6% which may be just a glimpse of bigger picture as in the present study only dominant population was studied. Selective media were not used for screening ESBL gut carriage which would reflect the true representation of ESBL carriage in the community. The low isolation of ESBL producers on D1 may be due to the short duration of exposure to the maternal flora during delivery (Table 2). Various factors could have contributed to the increase in the resistance by day 60. After delivery, exposure related to mothers environment, oral and skin flora provide the major sources of bacteria which may transfer to the neonates by several ways including suckling, kissing and caressing. In addition, breast milk is also a source of bacteria, which contains up to 10^9^microbes/L in healthy mothers [17]*.* Other sources may be household contact with siblings, pets [18], as well as horizontal transfer of gene within the commensal flora [1]*.* In our study acquisition of resistance via supplementary food has been ruled out as babies were completely breast fed. Several studies have shown the prevalence of antibiotic resistance in absence of direct use of antibiotic. Presence of tetracycline resistance bacteria in breastfed infants [19] and commensal ESBL producers in pre-school healthy children [20] suggest contamination in the family environment rather than direct exposure to antibiotic. The limitation of our study is that we have not studied the environmental flora and compared it with that of neonatal gut flora.

Besides ESBL, AmpC producing *Enterobacteriaceae* were also isolated. AmpC producing isolates were approximately 20% and co-production with ESBL was seen in 11.2% throughout the study period (Table 2). AmpC β-lactamases producers are of major concern as they are resistant to β-lactam and β-lactam inhibitor combination as well as cefoxitin which further narrows down the treatment options. As carbapenems are drug of choice for ESBL and or AmpC producing bacteria, coexistence of these enzymes can pose a threat to the community acquired pathogens as MIC of such strains are 10 fold higher for various carbapenems [21].

The *ampC* gene showed diverse profile, in contrast *CTX-M-15* was predominant *ESBL* gene in gut flora. Previous studies from India have also shown *CTX-M-15* as predominant ESBL from clinical isolate [22]. Approximately, 50% of neonates admitted to neonatal unit in our hospital with early onset sepsis had ESBL producing *Enterobacteriaceae*[23] which is strongly supported by early colonization with ESBL producing *Enterobacteriaceae* in the neonates in the present study.

Recent report of isolation of CRE (*NDM-1*) from environmental samples [9] and community acquired infections [24] indicate that CRE producing NDM-1 enzyme may be widely distributed in India. However, there is paucity of data regarding fecal carriage of CRE in the community in absence of antibiotic pressure. Different studies have used different culture based techniques like MacConkey agar plates supplemented with 1 μg/ml imipenem, Chrom Agar KPC, Mac Conkey Agar with imipenem, meropenem and ertapenem disc (10 μg) and two step selective broth enrichment method using 10 μg carbapenem disc to evaluate gut colonization with CRE with good performance [15]. Most of these techniques are validated for KPC detection in organisms with MIC range 0.5 - >32 μg/ml for various carbapenems [15]. Nordmann et.al. screened 27 *NDM-1* positive isolates and reported that the MIC of these isolates vary from 0.5 - >32 μg/ml, 1.5 - 231 >32 μg/ml and 1.5 - >32 μg/ml for ertapenem, meropenem and imipenem respectively. However, only one isolate i.e. P *Providencia rettgeri* A showed MIC of 0.5 μg/ml for ertapenem [25]. In present study with 2 step broth enrichment method using meropenem disc only one strain of *Enterobacter* sp was positive by MHT and PCR confirmed presence of *kpc-2* gene. MIC of other 28 suspected CRE isolates were ≤ 0.5 μg/ml for all carbapenems. Two isolates were positive for ESBL and AmpC, having MIC of 0.5 μg/ml for ertapenem but were negative for carbapenem genes.

In the present study widespread resistance to Ampicillin and 3rd generation cephalosporin (3GC) was observed but carbapenem resistance was rare. This can be explained by indiscriminate use of 3GC in human and animals due to availability of oral formulations and over the counter unrestricted access. Ampicillin and 3GC are used as an empirical therapy in India for the management of neonatal sepsis and other heath related complications like UTI, meningitis, bacterial sepsis (6, 1). The high prevalence of resistance to these drugs as indicated in our study raises the question regarding the efficacy of these antibiotics as an empirical therapy.

Carbapenems on the other hand are used sparingly as they are available as parentral formulation for which a patient have to visit the health care facility and in addition there is no reports of their use in animals from India. It is noteworthy that the presence of *kpc-2* gene in antibiotic naive neonates may be an alarming finding as carbapenem resistance genes are on plasmids and have a potential for rapid dissemination in future. Commensal flora can colonize the human gut without causing any symptoms, but most of the infections are endogenous and come from patient’s own gut flora [26].

The present study estimate of β-lactam resistance may be biased due to following reasons. Babies were supplemented with probiotics which have beneficial effect on gut by producing organic acids, bacteriocins, peptides and in turn decreasing pH of gut leading to inhibition of colonization of *Enterobacteriaceae*[27]. In addition, only the subdominant population was screened for ESBL carriage resulting in an under estimate of ESBL in the community. However, this data could not be an over-estimate as there are no reports of presence of *ESBL* genes in probiotic bacteria or transfer of antibiotic resistant genes from gram positive (Probiotic) bacteria to gram negative bacteria.

## Conclusions

Our data strongly suggest there is a tremendous load of ESBL and/or AmpC in the community in absence of any direct selection pressure indicating that these genes are widely distributed in the environment. This may result in significant increase in carbapenem use in community resulting in development of carbapenem resistance which may be due to porin loss with ESBL or de-novo spread of true carbapenemases. In conclusion there is a need to make efforts to determine the resistance load present in the different environmental pools (human, animal, and plants).

## Competing interests

The authors declare that they have no competing interests.

## Authors’ contributions

CK carried out all phenotypic work, DNA extraction, PCR, sequencing, and drafted the manuscript. RG conceived of the study and participated in its design, and edited the manuscript. LCS had done the analysis of the sequencing data. AS have designed the study. VK monitored the mother and the neonates for clinical outcomes and have trained the field workers. SA supervised the monitoring of the clinical outcomes. HC designed the clinical study and edited the manuscript. SS and MD had done the final editing and approved the final manuscript. All authors have read and approved the final manuscript.
